# Supplementary material for: Patterns of Intron Gain and Loss in Fungi
Source: PLoS Biol. 2004 Nov 30;2(12):e422. doi: 10.1371/journal.pbio.0020422 (PMC532390; doi:10.1371/journal.pbio.0020422)
Supplement: Table S1 — Also available at http://genes.mit.edu/NielsenEtAl/. (4.3 MB ZIP). [file pbio.0020422.st001.zip › NielsenEtAl/html/647.html]

AN7492.1.NCU06998.1.MG01422.1.FG09810.1


```
 CLUSTAL W (1.82) Multiple Sequence Alignments - Introns Inserted


Sequence 1: NCU06998.1	290 aa
Sequence 2: MG01422.1	323 aa
Sequence 3: FG09810.1	328 aa
Sequence 4: AN7492.1	326 aa
Alignment Length: 345 aa
Number Identitical Residues: 138 aa
Alignment Score (without introns) 7431


MG01422.1 	----MATTQEAEALVPKFKYERTLNQD1QAGRRTSLYGTIDSQPALVVLERASFPTS-AD
NCU06998.1	---MSDVKANAEALVPKFKFERQLNQD1QAGRRTTLLGTIDSSPALLILERAPFPSS-TD
FG09810.1 	---MADVKANAEALVPKFKLDRVLNQD1QAGRRISLYGSIDENPALLILERAPFSTS-QK
AN7492.1  	MDSNSGKKVAAEALISRFEITKLLKQD1QNGRRIVIQGSIDGQQGLLIAERAAFATESLA
          	 .:    .  ****:.:*:  : *:** * ***  : *:** . .*:: ***.*.:.:  

MG01422.1 	YLSSVTGTLRTLKNLGANDIYFWFMAATGG----------------TADAEDLKINLIYP
NCU06998.1	YLSSAPLTLRTLKNLGANDIYHWYMASSGGSSSSTNSNDPEKDHDEQTDFSDLKINLIYP
FG09810.1 	YLADVPAQLAQVQNLGANDIYHWYMGRSGAETTKP---------DDSDFFADLKINLIYP
AN7492.1  	VLKAFHSSITRIHNLGDNDIYRWYLASSGIDCDG-------------HQSHDLKLNVIWP
          	 *      :  ::*** **** *::. :* .                    ***:*:*:*

MG01422.1 	CTEQHVKKYSRQGVRFVVETPEIYKTKIRPYMQSKREAGRLSWVYNIIEGRKEVEDVIYR
NCU06998.1	CTPKHIAKYSRQLVRYVTETPALYQLHVRPYMQAQREAGRLNWVFNIIEGRKEVEDVIYR
FG09810.1 	CTEAHVKKYSKQGVRFVTETPEIYMNHIRPYMLSKREQGRLNWVFNIIEGRKEVEDVMYR
AN7492.1  	CTEQHIKKYSDQVLRMVTETPEIYRDYVRPYMQEKREEGRLNWVFNILEGRTEQEDVMLR
          	**  *: *** * :* *.*** :*   :****  :** ***.**:**:***.* ***: *

MG01422.1 	TPLG-EAGDEGFLLLPDLNWDRSTREALHLLGLVERRDIWSLRDLTKRHVSWLRHMRQRF
NCU06998.1	TPFGQDADDEGFLLLPDLNWDRKTLDALHLLGLVERRDLWSLRDLKKKHVPFLRHMKRKF
FG09810.1 	TKLG-EAGDEGFLMLPDLNWDRKTLDALHLLALVERRDIWSLRDLRKKHIPWLQHMKTRL
AN7492.1  	-DDG-HGAEDAFLMLPDLNWDRKTLGSLHLLALVHRRDLWSLRDLKKKHVPWLKYLRKRV
          	   * .. ::.**:********.*  :****.**.***:****** *:*:.:*:::: :.

MG01422.1 	LDATAEVYPEIERDQLKLYVHY~QPTYYHFHIHIVHVQLEAGATQATGKAVGLES~IIEQ
NCU06998.1	VEATTQVYPDIEEDQLKLYVHY~QPTYYHFHIHIVHVQLEAGATQAVGKAVGLDR1K---
FG09810.1 	IDATVKTYPSIEPDQLKLYVHY~QPTYYHFHIHVVHVQLEAGATQATGKAVGLES~IMET
AN7492.1  	LEGTVKMYPDLEEDQLKLYVHY1QPTYYHFHIHIVNVMLEAGATQATGKAFGLEN~LISQ
          	::.*.: **.:* ********* **********:*:* ********.***.**:    . 

MG01422.1 	LESMSGGDDAGMDLVSMGYTLGEASELWTEIFEPLKRETTSKPSTSAQ
NCU06998.1	------------------------------------------------
FG09810.1 	LGAMAGDDEAGMDGIAMSYTLGEASELWTEIFEPLKTGGEASQSQ---
AN7492.1  	LETLSGGEEASMADVSLTYYLGEASELWTEIYAPLKRGEKLAGK----
          	  : :....:.    :     ..::.  :.   . .       .
```
